# Introducing Peer-supported Open Dialogue in changing mental health care

**DOI:** 10.3389/fpsyg.2022.1056071

**Published:** 2023-01-18

**Authors:** Karin Lorenz-Artz, Joyce Bierbooms, Inge Bongers

**Affiliations:** ^1^Tranzo, Tilburg School of Social and Behavioral Sciences, Tilburg University, Tilburg, Netherlands; ^2^Mental Health Care Institute, Eindhoven, Netherlands; ^3^Erasmus School of Health Policy and Management (ESHPM), Erasmus University, Rotterdam, Netherlands

**Keywords:** peer-supported open dialogue, mental health care, severe mental illness, transformation, network-oriented approach, recovery-based approach, client-centeredness

## Abstract

The need to transform mental health care toward person-centered, recovery-based, and network-oriented care is recognized worldwide. Open Dialogue (OD) is seen as a hopeful approach in the context of this transformation and is introduced in countries around the globe. Five Dutch mental health care organizations spread over the Netherlands introduced the Peer-supported Open Dialogue (POD) approach, which adds an explicit role of peer-support workers to the OD approach. It appeared that (P)OD-trained professionals face issues in introducing the (P)OD approach in existing MHC settings. One of the reasons, which is the focus of this study, may be that they encounter difficulties in explaining to non-(P)OD-trained professionals what (P)OD entails. The main objective of this study is to provide guidance to and contribute to making (P)OD better understandable for non-(P)OD-trained professionals. In this study, we used a qualitative design and conducted 23 semi-structured interviews with POD-trained professionals with various backgrounds, to cultivate a rich understanding of which aspects could contribute to a better understanding of POD for non-POD-trained professionals. We used a hybrid approach to analyze the data, meaning that the technique of both inductive and deductive thematic analyses has been applied. From these analyses, six aspects emerged that could give guidance to and contribute to making (P)OD more understandable for non-(P)OD-trained professionals: (1) Experiencing (P)OD by attending treatment network sessions, (2) a coherent and profound narrative about (P)OD, (3) adjusting terminology to better fit the context, such as the two terms “principles” and “responsibility” in this study, (4) the order in which (P)OD elements are introduced in the narrative, (5) bringing the elements “presence,” “reflecting,” and “expertise by experience” more to the foreground, and (6) conceptualizing the main elements in a “talking paper.” A better understanding of (P)OD might be one of the building blocks for improving (P)OD adoption in existing MHC practices, which are on their way toward person-centered, recovery-based, and network-oriented care.

## 1. Introduction

At the moment, the necessary transformation of mental health care (MHC) toward person-centered, recovery-based, and network-oriented care is receiving increasing attention across the globe (Stupak and Dobroczyński, 2021; von Peter et al., 2021; WHO, 2021; Galbusera et al., 2022). This transformation entails a paradigm shift beyond the biopsychosocial model (diagnosis, medication, and symptom reduction) to a more holistic approach including an existential component (van Os et al., 2019; Galbusera et al., 2022) that conceptualizes recovery as a personally unique ongoing process encompassing all aspects of human life and concerned with gradually rehabilitating a sense of agency and meaning in life (Anthony, 1993; Slade et al., 2014). Subsequently, MHC practice should be based on equal collaboration between client, network, and care professionals to promote hope and empower people, shifting the focus from diagnosing and intervening to mobilizing the resources of clients and their closest network (“being with” instead of “doing to” people in distress; Seikkula, 2011; Slade et al., 2014; Schϋtze, 2015; Stupak and Dobroczyński, 2021; von Peter et al., 2021; WHO, 2021).

Open Dialogue (OD) is seen as a hopeful approach to this necessary transformation (Stupak and Dobroczyński, 2021; von Peter et al., 2021; WHO, 2021). The OD approach already embodies this needed change with its person- and network-oriented and recovery-based philosophy (Lakeman, 2014) and provides promising results in West Lapland (Finland), dealing with a severely acute mental crisis (Seikkula et al., 2006, 2011; Bergström et al., 2018). Seikkula (2003) explains that OD provides a rapid response to the crisis by meeting with the client and their closest network, preferably at people’s home, in an open dialogue network session within 24 h after contact. Professionals aim to generate dialogue, to create a new and shared meaning of experiences, and to empower people to take ownership of their desired changes. In addition, Hopfenbeck (2015) describes OD as a value-based practice, since OD explicitly describes its core values, including unconditional warmth, authenticity, and openness. In the literature, the approach is often explained by its seven guiding principles: (1) immediate help, (2) social network perspective, (3) flexibility and mobility, (4) responsibility, (5) psychological continuity, (6) tolerance of uncertainty, and (7) dialogism (e.g., Seikkula et al., 2011).

In this study, we focus on the introduction of the OD approach in a context of changing mental health care toward person-centered, recovery-based, and network-oriented care. In the search for better recovery-oriented care, five Dutch MHC organizations spread throughout the Netherlands introduced the Peer-supported Open Dialogue (POD) approach into daily ambulatory care for people diagnosed with severe mental illness. POD adds an explicit role of peer-support workers to the OD approach (Razzaque and Stockmann, 2016; Bellingham et al., 2018), referring to paid professionals with expertise by experience which means that they deploy experiential knowledge “gained through lived experience of psychological distress” (Bellingham et al., 2018, p. 1575). Moreover, POD embraces the adage “nothing about us, without us” (originating from the recovery movement), referring to the call for transparency (Hopfenbeck, 2015).

Organizations may encounter difficulties in translating a broad vision of a needed change into practice as Johansen et al. (2018) found in their “Expedition to Sustainable Healthcare.” This has also proven to be the case for OD. It appeared that OD-trained professionals face issues in introducing the OD approach in existing MHC settings, which complicates the adoption of the OD approach (Ong et al., 2019). Literature shows that to fully embed OD, as an approach that embodies the necessary transformation, the context—the existing MHC system as a whole—needs to change as well (Stupak and Dobroczyński, 2021; Von Peter et al., 2021; WHO, 2021). This requires a genuine understanding of what (P)OD entails, which oftentimes appears in practice to be hindered by the complexity of explaining the concept to non-trained mental healthcare professionals. This may be due to the lack of a widely accepted manual that comprehensively explains how OD is delivered (Buus et al., 2017, 2021; Waters et al., 2021). As a result, OD is often not considered as a new way of care (Søndergaard, 2009). The common and seemingly simple question “what do you do in OD?” from non-OD-trained professionals calls for a complex answer (Ong et al., 2019). This may be related to the question Seikkula (2011) raises: whether the OD approach is “psychotherapy” or a “way of life” (p.179). Ong et al. (2019) suggest reformulating the question to “how do you know that you are dialogic?” (p. 420), which allows room to distinguish between “doing” (psychotherapy) and “being” (way of life) in the answer to the question of what (P)OD entails. When introducing POD into Dutch MHC practice, Dutch POD-trained professionals indeed encountered difficulties in explaining the POD approach in an understandable and integrated manner to other professionals and stakeholders.

The main objective of this study is to provide guidance to and contribute to making (P)OD better understandable for non-(P)OD-trained professionals. A better understanding of (P)OD might be one of the building blocks for improving (P)OD adoption in existing MHC practices, which are on its way toward person-centered, recovery-based, and network-oriented care.

## 2. Materials and methods

This study was part of a broader study that aims to gain a better understanding of the introduction of POD in the Dutch (MHC) context. In this study, we used a qualitative design and conducted semi-structured in-depth interviews, to cultivate a rich understanding (Baxter and Jack, 2008) of which aspects could contribute to a better understanding of POD for non-POD-trained professionals. We used a hybrid approach to analyze the data, meaning that the technique of both inductive and deductive thematic analyses has been applied (Fereday and Muir-Cochrane, 2006). This study was approved by the Dutch Ethical Review Board of Tilburg School of Social and Behavioral Sciences, Tilburg University (REF RP195).

### 2.1. Setting

In 2017 and 2018, POD-trained professionals formed six POD networks spread over the Netherlands and introduced the Peer-supported Open Dialogue (POD) approach in five (MHC) organizations (Table 1). One of these networks turned an ambulatory team into a POD team (MHC organization in the southern part of the Netherlands, province North-Brabant), in which the POD approach and its network sessions were at the core of the care, in which other therapies were provided on demand. In this same organization, there was another POD network, in which POD-trained professionals worked in one ambulatory team together with other non-POD-trained professionals and provided day-to-day regular care. These POD professionals organized POD network treatment sessions on request and in addition to regular care. The other four POD networks were communities of POD professionals: one in the north, two in the center, and one in the southeast of the Netherlands. These POD professionals worked in regular teams spread over their (MHC) organizations and provided regular care with non-POD-trained colleagues. In addition to the day-to-day regular care, these POD professionals were connected within the POD community *via* WhatsApp, in which they organize couples for POD network treatment sessions. These POD sessions were organized on request of and in addition to regular care. The POD professionals delivered care to clients suffering from severe mental illness, who differed with respect to the living situation (at home, at an assisted living facility, temporarily admitted to crisis service) and registered diagnoses (e.g., depression, autism spectrum-, anxiety-, bipolar-, and psychotic disorders). At the point of data collection, over 90 POD-trained professionals have been striving to provide care within the Dutch MHC context based on the POD approach.

**Table 1:** Overview of POD networks and participants’ professional background per POD network.

	1. POD team	2. Within one ambulatory team	3. POD community	4. POD Community	5. POD community	6. POD community
*5* (*MHC*) *organization in the Netherlands*	The MHC organization in the south (province North Brabant)		Organization for guidance on key areas of life, in the center (province Utrecht)	The MHC organization in the center (province Utrecht)	The MHC organization in the southeast (province Limburg)	The MHC organization in the north (province Groningen)
*Manager*	1	1	2	1		
*Principal practitioner: Psychiatrist/Psychologist/Nurse specialist*	1	1		2	1	2
*Peer-Support worker*	1		1	1	1	1
*Case/career manager*	1			1	1	1
*Therapist/trainer*		2				

### 2.2. Procedure

#### 2.2.1. Participants

The first author asked the board of the national Dutch POD foundation, represented by the five organizations, to provide a POD-trained contact person for each POD network (six in total). These contact persons had coordinating roles in the implementation of POD and had a representative overview of the specific organization. They introduced the study and researcher to the POD professionals. With the intention to incorporate different perspectives, the researcher asked each contact person to list the names of POD-trained professionals working in their network, who had different professional backgrounds and preferably differed in the extent to which they support the POD approach. The researcher approached four POD-trained professionals per POD network.

Sampling was based on purposive (maximum variation in professional background and attitude toward POD) and convenience approaches (Ritchie et al., 2014). The 24 eligible participants received an information letter with the request to respond within 2 weeks. In case of nonresponse, the researcher sent a reminder, and the contact person contacted the eligible participant. We received a total of 23 eligible participants’ signed informed consent, after which the interviews were planned and conducted (Table 1). One eligible participant could not participate due to time constraints in the COVID-19 pandemic period.

All POD-trained participants attended the 1-year postgraduate training entitled “Peer-supported Open Dialogue, Social Network and Relationship Skills” at the Academy of Peer-supported Open Dialogue (APOD) in the United Kingdom, which is a course accredited at an English-speaking University at Post-Graduate Certificate level. The course consisted of four 5-day residential modules. Furthermore, the first author of this paper attended this one-year postgraduate training prior to this study.

#### 2.2.2. Semi-structured interviews

The first author conducted 23 semi-structured interviews for 60–90 min using zoom video conferencing during September and October of 2020. The interviewer showed the seven OD principles (Seikkula et al., 2006) and 12 key elements of Olson et al. (2014) on the screen during the interview. The research team had prepared a number of questions *a priori* for the interviews as an aid memoir for the interviewer, which included questions related to participants’ vision of the POD approach, e.g., the appropriateness and innovativeness of the OD principles, the adage “Nothing about me without me,” and the role of peer-support workers. For example, related to the first principle, the interviewer asked participants “Could you say something about what you think of the first principle?,” “How should the principle be applied according to you?,” “Why do you think that this principle is important?,” and “How does it match with client’s needs?” The participants were encouraged to share their views, regardless of whether they managed to apply them in practice at that time and to speak freely on aspects they considered relevant for the introduction of POD in practice.

### 2.3. Data analyses

All interviews were audio-recorded with permission of the interviewees, transcribed verbatim, and analyzed with the program Atlas.ti. These interviews were analyzed through Braun and Clarke’s six phases of thematic analysis, respectively: familiarizing with the data, coding, generating themes, reviewing themes, defining and naming themes, and producing the report (Braun et al., 2019). Prior knowledge is suspended as much as possible, also known as “bracketing” (Patton, 2014). The analysis was an iterative and reflexive process in which we used a hybrid approach, by using a codebook representing the seven POD principles (Seikkula et al., 2006), expertise by experience, and the adage “Nothing about me without me” (deductive) and adding new codes when encountered inductively as well (Fereday and Muir-Cochrane, 2006). The inductive part was related to both what participants want to communicate about POD and how they can best communicate (meta-communication) to make POD better understandable (Appendix I). During these analyses, we also compared responses between participants with different professional backgrounds and the POD networks to see whether responses were different among the disciplines and POD networks. This turned out not to be the case. Any doubts about the coding were discussed with a second researcher. The analyses were completed when no new themes emerged, and saturation was reached. These analyzes gave us insight into both the common ground for what POD participants found important to explain about POD to non-POD-trained professionals and what aspects they found useful to better explain POD (meta-communication).

## 3. Results

In this section, we outline the aspects, which emerged from the analysis process, which could contribute to making POD better understandable for non-POD-trained professionals.

### 3.1. A narrative about POD

Participants reported that both experiencing POD by attending POD network sessions and a narrative about POD as a handhold to explain POD could contribute to a better understanding of POD among non-POD-trained professionals. Interestingly, by reading the transcripts, we found a clear common ground in the individual meaning of (why important) and vision (what is “good care”) on the POD approach and what participants considered important ingredients to include in such a narrative for the Dutch context.

“*One thing that would help enormously is if at some point POD could be properly explained to people who are not POD-trained*, *so that there is more support*, *and more understanding of POD*. *And I think one way to make POD more understanding is if someone just joins a POD network session once*. *As network*. *Because I have noticed*, *for example*, *that because a colleague had once experienced such a POD session*, *she was better able to judge later whether a POD session could be useful*” (*POD-trained peer-support worker*).

In such a narrative, participants would consider the following as the main ingredients of POD: the dialogical process (principles “dialogism” and “tolerance of uncertainty”), including the involvement of the network with its multiplicity of perspectives (principle “social network perspective”), and the adage “Nothing about me without me.” Furthermore, they would describe the other organizational elements as valuable elements to improve the quality of treatment and to effect change. Moreover, participants would add that the principles of “flexibility and mobility,” “responsibility,” and “psychological continuity” should not be drawn into the absolute or regarded as limitless.

“*It’s not limitless…And here too*, *it plays a role again*. *You can also draw that into absolute*, *making it impossible to do anything with it*” (*POD-trained manager*)

In addition, participants mentioned that they would adjust the two commonly used terms within the (P)OD approach “responsibility” and “principle” for terms that better fit the Dutch context. First, they explained that the term “responsibility” can be confused with the commonly used term “principal responsibility” within Dutch mental health care, referring to a practitioner who is formally responsible for the treatment. Therefore, participants proposed using the term “involvement” instead. Second, they proposed to use the term “elements” as a more neutral term in Dutch instead of the word “principles,” as is the case in the “original” approach. They said that the word “principle” in Dutch can give the impression that it is a matter of principle with an obligatory character, and therefore, the risk of dogma is lurking.

### 3.2. Coherence and profoundness

Participants expressed that they experience difficulties in explaining the POD approach in a coherent manner and bringing to light the profoundness of the POD approach. By coherence, they meant the interrelatedness of the elements and layering of the approach itself. By profoundness, they meant the underlying theories and history of the approach and the notion that POD goes beyond learning new skills (the “doing” of dialogism) and also involves a personal change in the vision, values, and attitude of the professionals (the “being” of dialogism).

They said that when telling non-POD professionals about POD, they often use the seven (P)OD principles to explain what one is doing in POD because it provides a practical framework. However, they notice that it is not immediately obvious that (P)OD is based on certain values and entails a certain culture. They also explained that if you do not manage, in such a narrative, to convey this complexity to non-POD-trained professionals, misconceptions quickly arise. Their suggestion for a narrative would, therefore, be to also explain why something is done in a certain way.

“*There is such a deep great history*, *and there are actually all kinds of deliberate forms of therapies underlying it*, *but you do not see it directly in the principles*. *I also find that difficult when you explain what Open Dialogue is and you use the seven principles to make it clear that it is not just a conversational technique that you also learn in a course…Maybe that is what I miss in the POD principles*, *that POD requires another culture*” (*POD-trained principal practitioner*).

*“POD is of course a way of working*, *but it is not just a technique*. *It is a complete change of your whole being*. *As a human*. *So it has not only changed me in my work*, *but also in my general balance*, *being and contact and in terms of resilience…it really changed and helped me very positively*, *and I had not thought of that beforehand and did not expect it”* (*POD-trained case manager*).

These findings brought to light that the introduction of (P)OD to non-(P)OD-trained professionals summing up the seven (P)OD principles may not be enough to show the coherence within and profoundness of the (P)OD approach. In addition, three meta-communicative aspects emerged from the data analyses that might help to emphasize in such a narrative the coherence and profoundness of the (P)OD approach, to make it better understandable for non-(P)OD-trained professionals: (1) the order in which (P)OD elements are introduced in the narrative, (2) putting the elements “presence,” “reflecting,” and “expertise by experience” more to the foreground, and (3) conceptualizing the main elements in a “talking paper.”

#### 3.2.1. The order in which POD elements are introduced

In telling non-POD professionals about POD, participants tended to start by explaining the organizational elements, with the result that non-POD-trained professionals often stated that they already work that way. Therefore, participants considered it helpful to start a narrative by explaining the innovative core aspects of (P)OD followed by organizational elements.

“*Because when people talk about POD*, *the 7 principles are often used*, *but there is still a whole layer underneath*. *And if you only name those seven principles*, *you may interpret them completely differently because you interpret from a different starting point*. *That is of course what often happens now*, *when we talk about POD somewhere*. *That people often say that they already do that*. *And sometimes I can imagine that people say that*, *but then I still think*, *no! Your starting point is different*” (*POD-trained manager*).

Therefore, participants suggested that in such a narrative it matters in which order (P)OD elements are introduced and they proposed to: (1) start with the underlying theories and paradigm shift (the “why”), (2) continue with the adage “Nothing about me without me” (the “doing”), (3) explain the required attitude (the “being”), (4) elaborate on the required skills (the “being” and “the doing”), and finally (5) describe how to involve the network in combination with the other organizational principles (the “doing”). For each of these four parts, we elaborate on what participants considered important ingredients to explain in the Dutch context:

##### 3.2.1.1. Underlying theories and paradigm shift

The participants suggested that it is important to explicitly explain the underlying theories and paradigm shift (the “why”) that are new for non-(P)OD-trained professionals. Therefore, they suggested for the Dutch context to describe in such a narrative the underlying view of the (P)OD approach on mental problems, namely as a resonance in the interpersonal. They would describe in such a narrative that the problem underneath the request for help should be seen as a shared interactional problem instead of an individual problem. Additionally, they would explain that these problems are often related to a lack of connectedness and language, as a result of things that are difficult to say aloud.

“*Psychiatric…or problems are never problems of one individual*. *It is always a resonance in the interpersonal*. *There is always a network involved*” (*POD-trained principal practitioner*).

“*because you are never alone in a crisis*. *It is always an interaction with your environment*. *With involving the network you already take so much burden away from the person who can no longer bear it*” (*POD-trained peer-support worker*).

Another foundation they considered relevant to mention is that no one has a monopoly on the truth and to explain that truth is based on a shared meaning where everyone’s voice is equally important. By this, they would refer to the theory of social constructionism and the importance of polyphony. They would add that the source of both the underlying problem and the power to recover lies within the client and their network. They considered this foundation helpful to explain why they find it fundamental to collaborate with the client and their network, to (re)connect with and between the network members and to find shared meaning, instead of involving the network as a resource group to “solve problems.”

“*And which is really different*. *Very often in the past you have been approached as a network to solve other people’s problems*. *And POD is not about helping the other*, *it’s about everyone sitting there*” (*POD-trained manager*).

Participants conceived starting such a narrative with these foundations as important to clarify the rationale behind the proposition that connection and insights rather than consensus and solutions are considered the driving force behind change. Participants foresaw that this proposition entails a fundamental shift in Dutch mental health care since society expects mental health care to solve problems and current Dutch mental health care is also set up this way at the moment.

“*There are expectations from the mental health care that problems will be solved so that you no longer suffer from those problems*, *and that is not how POD is set up*” (*POD-trained principal practitioner*).

##### 3.2.1.2. The adage “Nothing about me without me”

The participants expressed that these underlying foundations and fundamental changes are embodied by and become tangible through the adage ‘Nothing about me without me’. Moreover, they said that this adage adds shared sense-making to the already familiar concept of shared decision-making, which ensures that the narrative would fit in with a concept that is already known. Therefore, they suggested continuing such a narrative with this adage because it could help to understand the implication (‘the doing’) of these fundamentals, e.g., abolition of multidisciplinary consultations.

“*If you would apply the adage for 100%*, *I have often said*, *you radically change mental health care*. *The ‘Nothing about me without me’ adage helps enormously to continuously involve people because you need them*” (POD-trained manager).

##### 3.2.1.3. The required attitude

According to POD professionals, such a narrative should then go into the basic attitude of (P)OD (the “being”) because it says something about what the foundations and adage imply for the professional himself and the way they connect and interact with the other. They used words such as “humble attitude,” “dropping the professional mask,” the importance of “unlearning,” and “the courage to be vulnerable in your profession.”

“*It’s not about them*, *it’s about us*. *If you want to change mental health care*, *you should not change the clients*, *but you will have to change the way you approach them*. *And that change is up to us*. *And the POD training provided for that*” (*POD-trained manager*).

In describing this basic attitude, participants would explain what is meant with and emphasize the importance of being authentic, present, and open in contact (“being with” instead of “doing to” people in distress). According to participants, this could help to explain to non-(P)OD-trained professionals how to shift from intervening expert to participating human being with experience and expertise. Participants would also describe the notion of unconditional warmth, referring to compassion and a full unconditional appreciation of the other because it differs from the common notion of distance and proximity.

“*There are many implicit assumptions about how to do POD right*. *‘Sitting with the family’ is a baseline for whether you have worked the POD way: have I really been there*, *have I really sat down next to people*. *Presence is the essence*” (*POD-trained principal practitioner*).

##### 3.2.1.4. The required skills

As a next theme, participants would elaborate on the required skills. They expected that these skills give an answer to the ‘doing’ of (P)OD and could show non-(P)OD professionals how to enhance connectedness between the client and their network and to create space in which hidden insights are given room to be unraveled (also called the unspoken). Participants found that the (P)OD elements dialogism and tolerating uncertainty best describe the needed core skills of professionals and that these skills belong together as reciprocal conditions.

With regard to dialogism, participants said this element could help to explain why and how to shift from a solution-oriented perspective to a relation-oriented perspective with polyphony as a core concept. They would explain that professionals’ prior focus should be on establishing connectedness and creating a safe culture of sharing. Additionally, they would explain that in order to do this, professionals need to be fully present and responsive to what is happening at the time, rather than having their own or preset agenda and taking the lead.

“*…because that’s where we catch ourselves time and again: we are or remain a kind of detective in the mystery of that misery that is presented*. *You want to know things as care professionals: how did it come about*. *You may be curious about what is happening and certainly you may ask to say a little more about what someone is saying*, *but you are responding to what is being said*, *and you are not looking for new information*. *You leave that detective role and step into the in-depth role*” (*POD-trained principal practitioner*).

In addition, participants would emphasize in a narrative that dialogism is also an end to foster dialogue between people, empower people, and effect change. So that it becomes clear that the shift from “doing” to “being” is not the same as doing nothing, which participants said was sometimes the concern of non-POD-trained professionals. Therefore, in a narrative, they would underline the importance of not taking too little responsibility in contributing to change under the guise of following the pace of the process, tolerance of uncertainty, and the not knowing. That is, professionals should take responsibility to assume their role in the dialogical process: listening to each other and mutual sharing during reflecting moments. They would add that the extent of sharing and the boundary of tolerating uncertainty are personally and contextually determined, that this is a timing matter and that finding this balance is a personal process.

“*For example*, *I admire Seikkula*, *who sits on the edge of his seat leaning forward toward people*. *I think that’s wonderful and I can sit like this*, *but I cannot react like him*. *When Kurti talks*, *I think ‘wow’*, *that is a lot of energy and she also shares personal things*. *Whereas*, *Jaakko never speaks about himself*. *And so we all have something good*. *And we should use that for God’s sake*. *You have to*. *So it has to be internalized and not become a trick (POD-trained principal practitioner).*”

Moreover, participants found it important that in such a narrative tolerating uncertainty is properly explained because this element shows the paradoxical approach of POD, and it gives guidance to non-POD-trained professionals on how to provide recovery-oriented care. They found that this element helps to explain how professionals, instead of trying to solve the problem, can better align to the clients’ and networks’ pace in the process, respond to and reflect on utterances of each person and create space so that client and network can take responsibility in their own recovery process. In doing so, often the solutions in the form of insights come naturally from the client and her/his network.

In addition, participants would highlight in a narrative the skill reflecting to explain dialogism and tolerating uncertainty to non-trained-POD professionals because this skill is more tangible and already common. They would then elaborate on the so-called reflection moment (referring to sharing reflections with a colleague in the presence of the client and network during a network session), which they considered innovative for the Dutch context. They found it important that the narrative includes an explanation of how to disclose appropriately (from the POD perspective). They would add that, with unconditional appreciation as a basic attitude, this would imply that professionals reflect on what resonates and emerges in them, allowing themselves to be affected more and share more personal experiences from an authentic vulnerability than professionals educated with distance and proximity might be used to.

“*Sharing* (*personal*) *things requires a certain authenticity*, *a certain modesty*, *a certain vulnerability*, *which is not easy for everyone*. *So even if people want to share that*, *do you dare to say that? Do you dare to be vulnerable*, *in your profession? And I mean really genuinely vulnerable”* (*POD-trained peer-support worker*).

“*There was a discussion about professional contact and POD contact*. *But I am a very professional POD’er*, *also just a person with experiences in life*, *which can be both part of the dialogue if they are at the service of the dialogue*. *Then I find that very professional*” (*POD-trained casemanager*).

##### 3.2.1.5. Involvement of the network in combination with the other organizational principles

Participants would end such a narrative with a description of the organizational (P)OD elements in which they would view the facilitation of the network sessions with (at least) two POD-trained professionals as the backbone of the treatment process, in which all necessary therapies can be integrated. In this part, they would describe the reasoning behind the notion to involve the client’s closest network from the beginning on. They would refer to, e.g., developing a well-established therapeutic relationship, broadening everyone’s perspective on the situation, supporting and engaging the network, and smoothing the process of getting back to life without getting bogged down in old patterns again. In this context of creating a safe collaborative culture, participants would explicitly mention the value of peer-support workers. They found that peer-support workers are often very sensitive and adept at bringing out the unspoken, putting it in words, and making people feel heard and seen.

*“The peer-support worker*, *I work with… I find that every time a gift to facilitate network sessions with her*. *She always knows how to press the right buttons*, *where I theoretically feel there is something there*, *but she does that so beautifully because she can also place her own emotional experience in it*. *They naturally get the role of a ‘confidant’ pretty quickly and that’s very nice”* (*POD-trained principal practitioner*).

Finally, to explicitly show (P)OD’s need-adapted philosophy, participants would explain the elements “flexibility and mobility,” “responsibility/involvement,” and “psychological continuity” similar to the original OD approach. With regard to flexibility, participants would explicitly stress the importance of being flexible related to time (duration and frequency) and content of the session, without being limitless (90 min on average). They would explain when the time for a session is too limited as they were used to, it is challenging to get out of the “chak-chak-chak-mode,” and professionals will tend to reach for solutions, may be more formal, more directing, and monological in contact. In terms of frequency, they would explain in such a narrative that at the end of a treatment session, it is determined through shared decision-making if and when the next meeting will take place instead of automatically scheduling sessions.

#### 3.2.2. Putting “presence,” “reflecting,” and “expertise by experience” more to the foreground

So, in addition to introducing the (P)OD elements in a certain order in a narrative, participants would also bring three elements more to the foreground in their communication about (P)OD: “presence,” “reflecting,” and “expertise by experience.” They suggested that the elements “presence” and “reflecting” should be brought to the foreground to give more meaning to the term dialogism. Moreover, participants said that these terms are familiar to non-(P)OD-trained professionals and may, therefore, help them to gain a better picture of what is meant and needed to be dialogical. They expected that this could help non-(P)OD-trained professionals to differentiate between dialogism and having a dialogue with someone, as we all engage in conversation with one another.

“*So dialogue as a word*, *as an element*, *has little appeal to the imagination*. *We all do*, *don’t we? And that’s right*. *Only within the Open Dialogue does it have its own meaning*. *Can’t we grasp that in that principle? The answer is probably no*. *And then ‘reflection’ is also an important word*. *To make the attitude explicit*.*”* (*POD-trained principal practitioner*).

Furthermore, they would propose to use the term “expertise by experience” in a narrative to express the role of peer-supported workers and the importance of the professional skill to share experiences in a proper way as peer humans, which applies to all professionals. The latter is the reason that participants would propose the term “expertise by experience” instead of “peer-support workers” in a narrative about POD because they suggested focusing on the expertise and not the expert role. However, they would position the element “expertise by experience” as an organizational element in this stage of development because they believed that in the current context the desired position of “peer-support workers” within treatment teams is not yet self-evident and needs to be organized.

#### 3.2.3. Conceptualizing the main elements

Finally, to show the coherence and profoundness of such a narrative, participants considered a visual “talking paper” helpful to make (P)OD better understandable for non-(P)OD-trained professionals. They would use such a “talking paper” as a communication aid to untangle, illuminate, and delve into the key elements of the POD approach, without losing sight of the coherence and profoundness of POD (the “being” and the “doing”). They suggest not only listing the elements but also visualizing the elements in an interrelated (coherence) layered (profoundness) constellation. (P)OD professionals could then talk through layer by layer in the order that they suggested for a narrative. In practice, participants emphasized that the appearance of and relationships between the (P)OD elements are not linear or disentangleable and that such a “talking paper” similar to a narrative could change over time.

*“I think then*, *assemble the big picture”* (*POD-trained principal practitioner*).

*“I think those seven principles are preconditions*, *while those three core elements about attitude*, *that’s actually how you should be as a human being*. *That goes deeper*. *But I do not know why it’s been pulled apart like that”* (*POD-trained manager*).

## 4. Discussion

The main objective of this study is to provide guidance to and contribute to making (P)OD better understandable for non-(P)OD-trained professionals. There is rich literature about the OD approach and its underlying foundations (e.g., Seikkula and Trimble, 2005; Seikkula and Arnkil, 2006; Seikkula, 2019). In addition, studies refer to the potential risks of misconceptions about OD (e.g., Søndergaard, 2009; Ong et al., 2019; Waters et al., 2021), which is also recognized in practice. However, little is known about how (P)OD professionals can best explain the approach in practice to non-(P)OD-trained professionals to increase the understanding of (P)OD.

We found six aspects that could provide guidance to and contribute to making (P)OD more understandable for non-(P)OD-trained professionals: (1) Experiencing (P)OD by attending treatment network sessions, (2) a coherent and profound narrative about (P)OD, (3) adjusting terminology to better fit the context, such as the two terms “principles” and “responsibility” in this study, (4) the order in which (P)OD elements are introduced in the narrative, (5) bringing the elements “presence,” “reflecting,” and “expertise by experience” more to the foreground, and (6) conceptualizing the main elements in a “talking paper.”

One of the main suggestions in this study is that it can be helpful to consciously introduce the (P)OD elements in a certain sequence, in order to make (P)OD more understandable: starting with the underlying theories and fundamental view on mental health problems, continuing with the adage “Nothing about me without me,” followed by the required attitudes and skills, and finally the involvement of the network in combination with the other organizational elements. The POD professionals in this study indicated that it is tempting to use the seven OD principles as a quick start guide to introduce the POD approach to non-POD-trained professionals since it makes the profound multilayered approach more tangible and demarcated. Literature shows that these principles have been used to evaluate OD practices as well (Waters et al., 2021). However, the developers of the principles classified the principles as guidelines and did not intend to define OD (Seikkula and Arnkil, 2006). Similarly, using this list of principles as a backbone to explain (P)OD may not do justice to the coherence and profoundness of the approach. Consistent with Ong et al. (2019), the results of this study show that in order to make (P)OD better understandable a narrative about (P)OD would need to touch upon both the deeper layer (the “why” and the “being”) and the practical side (the “doing”) of (P)OD.

This study proposes to start such a (P)OD narrative by explaining its underlying theories and fundamental different view on mental health problems. (P)OD addresses the question of the etiology of mental disorders. Seikkula (2019) proposes that the human mind could be viewed as relational and subsequently, human behavior could be considered as part of the responsive relational context instead of attributed to a single person. Following this line of thinking, Stupak and Dobroczyński (2021) describe in their study that psychiatric disorders could be seen as a primary consequence of living conditions and their significance for individuals. Without delving into a discussion about which etiological view on mental disorders is the right one, continuing the (P)OD philosophy, opens new perspectives and leads to different questions, e.g., “what is wrong with you?” shifts to “what’s happened to you?” (Longden, 2013). Like in the metaphor that mankind once believed in the flat Earth model, their belief in a spherical Earth led to different questions and made questions such as “how far do I sail before I fall off the earth?” redundant (Bill, 2001). In other words, one can see the parallel that fundamental changes in thinking about our existence lead to new perspectives, different lenses to look through, and a set of new questions.

As non-(P)OD-trained professionals look through these new lenses, it may be clearer that the collaborative nature of (P)OD sheds new light on network-oriented care. Seikkula (2021) suggests that the novelty of the current MHC context may be that this therapeutic relationship and the importance of connecting and reconnecting people is not an aspecific factor within POD, as it is usually considered, but could be seen as the specific working factor of the approach. Understanding the “earth-is-round” suggestion of shifting from a solution-oriented perspective to a relation-oriented perspective could help to understand why POD’s primary intention is to connect and re-connect through a mutual process of uttering and responding, meaning, and understanding and giving words to the unspoken, without striving for consensus (Seikkula and Trimble, 2005). In addition, it may become clearer for non-POD-trained professionals that the approach requires a fundamental change of the professional him/herself, by turning from an expert trying to solve an issue or crisis (do to) to a human sitting with the client and his/her closest network (being with) and foster dialogue (Seikkula and Trimble, 2005). In a similar vein, the closest network is also not involved to solve something. This shift may help professionals to profoundly understand how recovery-oriented care can be put into practice (Damsgaard and Angel, 2021) and confirms the importance of the therapeutic relationship, connectedness, and integration, which is broadly considered crucial (van Os et al., 2019; Seikkula, 2021; Finsrud et al., 2022).

This study proposes to continue such a narrative with the adage “Nothing about me without me” as a tangible statement, which could be seen—from a POD perspective—as an embodiment of the “earth-is-round” fundamental for the current MHC context. For example, this adage takes shared decision-making, which is being pursued in current practice, to a higher level by extending it to shared meaning-making and shared decision-making (von Peter et al., 2019). This adage also demonstrates that an “earth-is-round” way of thinking can imply a radical reshaping of the current MHC, which is in line with the study of Beeker et al. (2021). That is, if this adage is fully applied, the consequence could be for example that multidisciplinary consultations behind closed doors are abolished.

This study suggests continuing, after this adage, with the required values, attitude, and skills because the associated elements relate to the dialogical mindset (Ong and Buus, 2021). Subsequently, after setting the scene by introducing first the “earth-is-round” fundamentals from a (P)OD perspective and this dialogical mindset, the other organizational elements are put forward. By introducing the central elements in this order, the organizational elements are considered through these new lenses instead of from the traditional point of view. This can help non-(P)OD-trained professionals differentiate the (P)OD approach from other integrated care models with familiar organizational elements (Von Peter et al., 2021). This order in introducing (P)OD elements differs from most literature on the POD approach, where the organizational elements are often presented first, and then it becomes clear in the explanation of the approach that the dialogical process is central (e.g., Seikkula et al., 2003; Seikkula and Trimble, 2005; Olson et al., 2014; Razzaque and Stockmann, 2016; Seikkula, 2021).

However, leaving the organizational elements until the last part of such a (P)OD narrative may also involve a risk of misconceptions. As Von Peter and Zinkler (2021) refer in their paper, there is a risk that others may believe that (P)OD is possible without realizing institutional reorganization and a risk that (P)OD may be used as a cloak to cover the current symptom reduction based system, without fundamentally changing it. Furthermore, Beeker et al. (2021) state that (P)OD requires a radical reshaping of the current MHC. This study suggests explaining, in such a narrative, how (some) organizational elements should be seen as prerequisites to do full justice to the adage, required attitude, and skills. However, it is still a risk to be aware of. Moreover, Ong et al. (2019) describe in their paper that these organizational elements can be seen as “operational” elements and can also help explain how one can know whether someone is “doing” POD. This could be another reason not to make the sharing of organizational elements in a POD narrative too small.

In line with the literature, the results of this study suggest that explaining the (P)OD approach only partly or fragmented in separate elements may result in a lack of genuine understanding of the (P)OD approach and dilute the uniqueness and innovativeness of the (P)OD approach (Søndergaard, 2009; Seikkula, 2021; Waters et al., 2021). For example, studies illustrated that the term dialogism is often reduced by professionals working within the existing MHC system to a communicative function, lacking the creative collaborative reciprocal act of finding new meanings and the notion of “being dialogical” (Seikkula and Trimble, 2005; Ong et al., 2019; Seikkula, 2021). This was also found in this study, which led to the suggestion to bring the elements “presence” and “reflecting” to the foreground, to give more meaning to the term dialogism. In addition, this study suggests conceptualizing the (P)OD approach in a “talking paper” to help to see fragments in coherence and as a backbone for a (P)OD narrative. This “talking paper” could be seen as a visual metaphor that presents several core elements in a layered interactively integrated constellation, rather than in a list (e.g., Seikkula et al., 2006; Olson et al., 2014; Razzaque and Stockmann, 2016). Such a visual metaphor could show in which order the elements can be best introduced in a (P)OD narrative and emphasize that these elements are mutually connected and entail both the “being” and the “doing.”

We hope that these six aspects give guidance to and contribute to making (P)OD more understandable for non-(P)OD-trained professionals. In doing so, we hope that this better understanding might be one of the building blocks for improving (P)OD adoption in existing MHC practices, which are on their way toward person-centered, recovery-based, and network-oriented care.

### 4.1. Study limitations and future research

We acknowledge that the study also has a number of caveats. One relates to the sampling, in which care was taken to include multiple perspectives of the POD-trained professionals to gain a rich view. However, we only took into account different professional backgrounds and their attitude on (P)OD. Other possible influencing factors were not taken into account, e.g., level of communication skills, degree of experience with applying (P)OD, or communicating about (P)OD. In addition, none of the participants was decidedly negative in their vision of POD. This may have skewed our participants’ view on what is needed to make (P)OD better understandable. Furthermore, the consequences of the COVID-19 pandemic may have influenced the choice of eligible POD professionals to participate in this study.

Moreover, these six aspects to provide guidance to and contribute to making (P)OD better understandable may be context-dependent. For example, the order in which the elements can best be introduced in the narrative can differ per context. If, for example, in a context the notions of involving the network, visiting clients in the home situation, or staying involved are new, it might be needed to introduce these elements earlier in the narrative than portrayed in this study, which took place in the Dutch context.

Furthermore, the aim of this study was to provide guidance to and contribute to making (P)OD better understandable for non-POD-trained professionals. However, the saying ‘it takes two to tango’ applies also to making (P)OD better understandable. In this study, we have only included the perspective of POD professionals. Whether the found aspects can truly contribute to making (P)OD better understandable should also be viewed from the perspective of non-POD-trained professionals. An example of an aspect that might be difficult for (P)OD professionals to judge is whether the language they use is attuned to the context. Moreover, the way it will be perceived may also be influenced by the tone of voice and the manner in which the message is conveyed by the (P)OD-trained professional. Introducing such a transformative philosophy requires an understanding, attentive, and careful approach. The (P)OD approach may provide guidance on how to convey the POD approach to non-POD-trained professionals. Analogous to the transformative dialogue with clients and the network during treatment sessions, POD professionals could entice non-POD-trained professionals to join in a creative collaborative reciprocal act of finding new meanings. First, the POD professional would then aim to connect with colleagues by being responsive to the utterances of the non-POD professional, and second, to gradually introduce the POD philosophy in an active participatory manner. Being dialogical could help to carefully consider the socio-cultural fit to local conditions (Buus et al., 2017) by being adaptable and responsive to the needs of the current MHC context (Ong et al., 2019).

The next valuable step after this study may be to evaluate with non-(P)OD-trained professionals whether and how the six aspects improve the understanding of (P)OD among non-(P)OD-trained professionals. The moment that (P)OD is better understood, the question will rise whether this better understanding indeed leads to greater support for (P)OD and to better adoption. Because even when (P)OD is fully understood and embraced, applying it in practice is another matter and requires nuance, timing, and balancing. For example, professionals should not draw the elements of the (P)OD narrative in absolute, which may be the risk of trying to capture (P)OD in a compact, comprehensive, and demarcated (P)OD narrative, just as Waters et al. (2021) refer to the risk of manualization of (P)OD. In practice, the appearance of and relationships between the (P)OD elements are not linear or sequential and disentangleable. For example, in applying (P)OD in practice, practitioners may encounter a continuous tension between two stances: one is the tendency to be humble and adapt to others’ needs and the other one is the importance of taking an active participating role in the reciprocal dialogical process. These conflicting needs do not need to be mutually exclusive but do require continuous balancing (Galbusera and Kyselo, 2019). This relates to the statement that a person-centered approach is per definition an interperson-centered and dialogical approach (Galbusera et al., 2022). This brings us back to the underlying POD notion that all voices equally matter (Seikkula and Trimble, 2005). So, it would be interesting to gain insight into other prerequisites – besides a better understanding—to further adopt and subsequently embed the approach in a changing (MHC) context toward person-centered, recovery-based, and community-oriented care.

Even though there are still questions to be tackled on the road to broader adoption of (P)OD, starting with making the POD approach better understandable for non-(P)OD-trained professionals, could be the first step to facilitating an open dialogue about the potentials of this approach within a changing mental health system on its way to (inter)person-centered, recovery-based, and network-oriented care.

## Data availability statement

The datasets presented in this article are not readily available because the raw data supporting the conclusions of this article cannot be made completely anonymous. Requests to access the datasets should be directed to KL-A, c.a.g.lorenz@tilburguniversity.edu.

## Ethics statement

The studies involving human participants were reviewed and approved by Ethical Review Board of Tilburg School of Social and Behavioral Sciences, Tilburg University (REF RP195). The patients/participants provided their written informed consent to participate in this study.

## Author contributions

KL-A, JB, and IB: conceptualization and methodology, and writing, reviewing, and editing. KL-A: thematic analysis and writing the original draft preparation. KL-A and JB: analysis and interpretation of results and project administration. JB and IB: supervision. All authors contributed to the article and approved the submitted version.

## Conflict of interest

The authors declare that the research was conducted in the absence of any commercial or financial relationships that could be construed as a potential conflict of interest.

## Publisher’s note

All claims expressed in this article are solely those of the authors and do not necessarily represent those of their affiliated organizations, or those of the publisher, the editors and the reviewers. Any product that may be evaluated in this article, or claim that may be made by its manufacturer, is not guaranteed or endorsed by the publisher.

## Abbreviations

POD: Peer-supported open dialogue
OD: Open dialogue
MHC: Mental health care
