# Supplementary material for: Introducing Peer-supported Open Dialogue in changing mental health care
Source: Front Psychol. 2023 Jan 18;13:1056071. doi: 10.3389/fpsyg.2022.1056071 (PMC9891459; doi:10.3389/fpsyg.2022.1056071)
Supplement: Supplementary file 1 [file Data_Sheet_1.PDF]

Appendix I: ‘Codebook’

Codes		Themes
**Deductive**	**Inductive**	
Immediate help		Organizational element
Social network perspective		Organizational element
Flexibility and mobility		Organizational element
Responsibility		Organizational element
Psychological continuity		Organizational element
Tolerance of uncertainty		Skills
Dialogism		Skills
Polyphony		Skills
Expertise by experience		Organizational element
Nothing about me without me		Fundamentals
	Foundations	Fundamentals
	Reflecting	Skills
	Presence	Value/attitude
	Authenticity	Value/attitude
	Openness	Value/attitude
	Unconditional warmth	Value/attitude
	Coherence	Meta-communication
	Profoundness	Meta-communication
	Fragmented	Meta-communication
	Experiencing POD	Meta-communication
	Different starting point	Meta-communication
	Language	Meta-communication
	Visual	Meta-communication
